# The Role of High-Mobility Group Box-1 and Its Crosstalk with Microbiome in Rheumatoid Arthritis

**DOI:** 10.1155/2017/5230374

**Published:** 2017-10-23

**Authors:** Federico Biscetti, Andrea Flex, Stefano Alivernini, Barbara Tolusso, Elisa Gremese, Gianfranco Ferraccioli

**Affiliations:** ^1^Division of Rheumatology, Fondazione Policlinico Universitario “A. Gemelli”, Institute of Rheumatology, Catholic University School of Medicine, Rome, Italy; ^2^Division of Internal Medicine, Fondazione Policlinico Universitario “A. Gemelli”, Institute of Internal Medicine, Catholic University School of Medicine, Rome, Italy

## Abstract

Rheumatoid arthritis (RA) is a chronic, definitely disabling, and potentially severe autoimmune disease. Although an increasing number of patients are affected, a key treatment for all patients has not been discovered. High-mobility group box-1 (HMGB1) is a nuclear protein passively and actively released by almost all cell types after several *stimuli*. HMGB1 is involved in RA pathogenesis, but a convincing explanation about its role and possible modulation in RA is still lacking. Microbiome and its homeostasis are altered in patients with RA, and the microbiota restoration has been proposed to patients with RA. The purpose of the present review is to analyze the available evidences regarding HMGB1 and microbiome roles in RA and the possible implications of the crosstalk between the nuclear protein and microbiome in understanding and possibly treating patients affected by this harmful condition.

## 1. Introduction

Among the autoimmune diseases, rheumatoid arthritis (RA) represents one of the most relevant [1, 2]. In fact, patients affected by RA have a poor quality of life, due to articular pain and functional impairment [3–7]. In addition, RA causes an increased risk of other pathological conditions, including cardiovascular diseases [8–16]. Furthermore, immunosuppressant for RA can often determine dangerous and potentially lethal side effects, among which are infections, organ failure, and even death [7, 17–24]. Although RA has been studied over the last decades and several researchers have been focused on identifying new potential drugs, a definite treatment is not available and the disease can progress to severe disability [4, 7, 17, 19, 25–30]. One of the reasons of the delayed defeat of the disease is the lack of a full understanding of the causes responsible for the RA onset. Indeed, while several pathways and mechanisms have been clarified, such as lymphocyte, interleukin, and tumor necrosis factor (TNF) roles, the very initial trigger has not been discovered [1, 23, 27, 28, 31–41]. As in other autoimmune conditions, an infectious event has been proposed to explain the altered immune response and the RA initiation [42]. In this scenario, microbiome obviously represents an attractive candidate. In fact, the altered crosstalk between microbiome and the immune system could underlie the disease onset [43–54]. Among the well-known pathways, the high-mobility group box-1 (HMGB1) plays a role in RA. In fact, this nuclear protein is involved in synovial inflammation observed in RA and could represent a new therapeutic target [55–68]. Recent data demonstrated that the HMGB1 pathway is important in a model of bowel inflammation [69]. The aims of the present review are to evaluate the available data about the role of HMGB1 in the crosstalk between gut microbiome and RA-altered immune response, to try to better understand the mechanisms underlying this disease, and to see whether it could represent a therapeutic target and, eventually, whether it would be more cost-effective to inhibit or stimulate the activity of HMGB1 in these conditions.

## 2. Rheumatoid Arthritis

RA is an autoimmune disease, characterized by chronic inflammation of joints and several other tissues, including those of the lungs, vessels, blood, eye, skin, and heart [70, 71]. RA is not a rare disease; in fact, out of every 100,000 people, about 40 are diagnosed with RA every year [72]. In general, at the onset, RA affects the small joints of hands. However, also hips, shoulders, and knees may be involved, and RA can potentially hit every joint [25, 33, 73]. Quality of life of patients affected by RA is worsened by pain, swelling, stiffness, and loss of function in the joints [4, 5, 74]. Furthermore, patients with RA have a reduced life expectancy due to an increased mortality for cardiovascular events, infections, and drug side effects [9–11]. In fact, it has been definitely demonstrated that patients with RA have an increased risk of myocardial infarction and of stroke [75]. The principal reason is that the typical chronic inflammation observed in the RA scenario plays a pivotal role in atherosclerotic plaque formation and destabilization [10, 11, 76, 77]. In addition, the immune dysregulation of T and B cell network can affect other cardiovascular risk factors, such as hypertension and lipid metabolism [9, 78, 79]. Furthermore, sedentary lifestyle and weight gain due to joint impairment could be additional factors. Other morbidity causes are certainly infections. In fact, immunosuppressant therapy and RA itself increase the risk of infectious complication, and about a quarter of deaths are caused by infections [42, 80–86]. Finally, several of the most effective treatments commonly used in patients with RA can have many side effects, including organ failure, cancer and, sometimes, death [18–20, 22].

Although the relevance and the impact of RA are clearly important, an effective treatment has not been yet discovered. The reason of this delay may reside in the relatively unknown initial pathological event. Indeed, several mechanisms have been clarified to explain the fundamental injury: the synovitis and the joint destruction [3]. First, a genetic susceptibility is known. In fact, an association between RA onset and major histocompatibility complex (MHC) class II antigens, specifically the shared epitope found on HLA-DRB1, has been demonstrated [3, 70, 71, 87–89]. However, RA does not seem to be a genetically transmitted disease, and DNA in the strict sense plays a minor role. Regarding the genetic heritage and regulation, novel mechanisms have been elucidated in the last decade, in particular the epigenetic regulatory systems, including the microRNA (miRNA) pathways [41, 90, 91]. Moreover, miRNAs can regulate gene expression and protein function of several cytokines, growth factors, and receptors involved in RA [41]. Alongside the genetic susceptibility, a trigger is required to initiate RA; in fact, studies performed on twins have demonstrated that identical genetics are not sufficient to develop similar disease [92]. Several potential environmental triggers have been implicated, among which are cigarette smoking and infections [93–95]. Taking into account infectious event, the relationship between RA and infective disease is dependent on the immune and inflammatory activation caused by pathogens [42, 96–98]. The T and B cell activation and the beginning of the autoimmune response are the mechanisms involved in the RA onset [32, 79, 99–103]. Another important event is represented by the protein citrullination, a normal posttranslational modification required in several physiological processes [104–107]. In RA, there is an autoimmune activity against citrullinated peptides detected as anti-citrullinated peptide antibodies (ACPA), a prototypical biomarker of the disease. After T and B cell activation and autoantibody production, additional cell types come into play to propagate and amplify inflammation, among which are macrophages that produce interleukin- (IL-) 1, IL-6, IL-8, and tumor necrosis factor- (TNF-) *α* [108–113]. All these phenomena translate into the main event of the disease: joint damage.

## 3. High-Mobility Group Box-1

The high-mobility group box-1 (HMGB1) is a highly conserved DNA-binding protein, present in the nucleus, that acts as a damage-associated molecular pattern (DAMP) molecule [114]. HMGB1 belongs to the family of the high-mobility group (HMG) chromosomal proteins, distinguished on the basis of their rapid mobility on electrophoresis gels [115]. These nuclear proteins were discovered more than 40 years ago and are subdivided into three superfamilies: the HMGB, HMGN, and HMGA superfamilies [116]. Of the HMGB family that includes HMGB1, HMGB2, HMGB3, and SP100HMG, HMGB1 is the most abundant nonhistone DNA-binding protein [114]. HMGB1 is the typical DAMP molecule, and it is involved in the setting of both sepsis and sterile inflammation [114]. This nuclear protein belongs to the “alarmin” family, a group of signaling effectors that acts as an injury-induced response in mammals [117]. DAMPs interact with several ancestral receptors and pathways and share a significant number of signaling systems with the pathogen-associated molecular patterns (PAMPs) [118]. DAMPs and PAMPs can activate the immune system by using the same ways, starting from completely different pathological triggers. In this scenario, HMGB1 represents the prototypical molecule that can stimulate a lot of immune responses against external injury. In this sense, HMGB1 could be considered exclusively a defensive protein. However, this protein plays also a dangerous and harmful role in numerous conditions by activating detrimental pathways so that many authors suggest the blockade of its function [119–121]. The role of HMGB1 in normal and in disease conditions was originally attributed to the passive release in the extracellular space after the cell damage [122]. Subsequently, a more complex mechanism of action was identified for HMGB1: it is also actively secreted by almost all types of cells, in response to several stimuli, and it can activate different pathways, depending on the tissue where the signaling is triggered and on the kind of receptor involved [118, 123]. The most recent findings have highlighted that the effect of HMGB1 is also closely dependent on the redox status of the milieu where the protein is released [124].

The first information about HMGB1 activity has been collected in models of sepsis and systemic infections [125]; the idea that this alarmin is involved in the sterile inflammation and fibrosis rapidly increased [55, 114, 117, 126] and fibrosis [127]. During the last decade, additional data were collected regarding more variegated effects of this nuclear protein in terms of tissue remodeling and angiogenesis, not necessarily related to septic conditions [115, 128–131].

## 4. High-Mobility Group Box-1 and Rheumatoid Arthritis

There are several data supporting the role of HMGB1 in RA, particularly suggesting that it plays a role in initiating the synovium inflammation and in maintaining the joint damage mediated by proinflammatory cytokines. Since the first studies by Andersson and coworkers, it has been clarified that HMGB1 can stimulate the release of IL-1, IL-6, and TNF-*α* [122] and it determines the beginning and the development of inflammation in different experimental models of arthritis. Furthermore, HMGB1 is increased in synovium and synovial fluid of patients with RA, compared with patients with osteoarthritis [132, 133]. Moreover, HMGB1 blockade reduces arthritis induction in experimental models [55, 56, 59, 63, 67, 134, 135]. Finally, HMGB1 administration induces synovial angiogenesis through a vascular endothelial growth factor- (VEGF-) dependent mechanism [55]. Although multiple mechanisms involved in RA pathogenesis have been discovered, there is no fully comprehensive explanation about the HMGB1 pathway in this scenario. In particular, HMGB1 function depends on two principal factors: the oxidation/reduction status and the extracellular milieu where different receptor systems can be found. While the second point is enough studied and we know now that the TLRs, the receptor for advanced glycation end-products (RAGE) and the IL-1 receptor, represent the most important extracellular pathways [61], we less know about the factors that modify the oxidation/reduction status of HMGB1. In fact, depending on oxidation/reduction status, HMGB1 can be in three different conformations: sulfonic, disulfide, or all-thiol form [58, 136, 137]. According to the redox status and following different structures, HMGB1 explicates various functions. For instance, the sulfonic form acts as an immune tolerance inducer, while the disulfide one is a major player in inflammation. In this sense, the HMGB1 pathway is notably plastic and dynamic and depends on the redox status of the extracellular setting, not only on the receptor quality and content [61]. However, it is not yet clear how the environment can modify the redox state and what cell types are involved in this process.

## 5. Microbiome

The term microbiome refers to the genetic characterization of the entire microbiota in a specific tissue [138]. We know several microbiomes, depending on localization, such as skin, lung, and oral microbiomes [139]. Certainly, the gut microbiome is one of the most important because, together with activities shared with other microbiomes, it plays a fundamental role in digestion and transformation of food [43, 45, 51, 140, 141]. However, the principal function of microbiome is the crosstalk with the immune system to modulate and regulate the immune response against the host. Gut is colonized by billions of bacteria immediately after birth, and the mucosal interface of the intestinal tract is characterized by several types of immune cells and systems, organized in aggregates and organs [140]. The location of these systems is strategically at the border with the outside world, and they require a multipotent and versatile network of signals and receptors. In fact, there we have the pattern recognition receptors (PRRs), an ancestral part of the immune system that can recognize several pathogens with the same pathway [142]. Among PPRs, toll-like receptors (TLRs) are the prototypical receptors that bind elemental fragments of bacteria, such as lipopolysaccharides (LPSs), and also of microbiota [142, 143]. However, given the number and the different types of species of gut microbiome, it seems unlikely that these bacteria activate the immune response normally. Most likely, the interaction between microbiome and intestinal immune system determines a continuous modulation of the two players [43, 144].

## 6. Microbiome and Rheumatoid Arthritis

The connection between gut and joints was hypothesized several decades ago, when researchers studied different models of inflammatory arthritides, in particular spondyloarthropathies related to inflammatory bowel diseases and secondary to intestinal resections [49]. The interaction between genetic profile and environmental triggers is important in the pathogenesis and development of RA. Oral chronic colonization or infection sustained by *Porphyromonas gingivalis* was linked to RA development [145, 146], and traces of bacteria were found in synovial fluid of patients with RA. Furthermore, prolonged antibiotic therapy against certain bacterial infections is effective in RA disease control [147]. Breaking tolerance in RA could occur in reaction to these pathogens. However, *Porphyromonas gingivalis* is not the only implicated in RA. In fact, data regarding other bacteria are available, and a single infection seems to be not likely as the sole cause. Moreover, the analysis of microbiome from mice prone to arthritis development revealed that microbiome can influence the arthritis susceptibility [148]. Several reports demonstrated that a subpopulation of patients with early RA harbored intestinal microbiota dominated by *Prevotella copri* and that SKG mice harboring the same microbiota had an increased number of intestinal Th17 cells and developed severe arthritis due to autoreactive T cells [149]. Interestingly, a taxon-level analysis-based study revealed an expansion of rare taxa with a decrease in abundant taxa in microbiome of patients with RA, compared with controls; this finding was related to the production of proinflammatory cytokines, such as IL17 [150]. Microbiome alterations do not only affect the expression level of TLRs of cells that exhibit antigens but also contribute to the Treg/Th17 deregulation. Epigenetic modifications triggered by external factors are important pathways leading to an altered gene expression. Crosstalk between microbiome and the mucosal immune system has been demonstrated being a crucial activator of epigenetic pathways in mammalian, including humans [43]. The most compelling evidence that gut infection-inflammation is a key moment in the occurrence of arthritis comes from the K/BxN and IL1RA−/− mice that do not develop arthritis in a germ-free setting [151]. On the other hand, the evidence that a normal gut microbiota is fundamental in maintaining the homeostasis is shown in the streptococcal cell wall arthritis, in which the normal flora protects against the occurrence of arthritis [152].

RA is a chronic multifactorial autoimmune disease where the immune event represented by the ACPA production can start even 15 years before symptoms, thus suggesting that the initial pathogen phenomenon is not necessarily present in the joints. In this scenario, microbiome represents the ideal theater [44]. Starting from animal models, about forty years ago, several researchers found that the administration of specific bacteria fragments, such as LPS, can induce arthritis and that the presence of gut microbiota is protective against the injury. Furthermore, additional evidence suggested that the balance of the intestinal germ population is fundamental in maintaining homeostasis and protection against environmental pathogens [153]. Recent data demonstrated that alteration of the gut microbiome can influence the balance of pro- and anti-inflammatory immune cells, such as T reservoir, and promote the development of RA [154]. Moreover, it has been found that TLRs play a crucial role in influencing the Th17 differentiation and the Treg inhibition caused by gut microbiome in animal and human models [142]. However, although a lot of possible mechanisms have been elucidated to demonstrate the role of microbiome in RA, a definitive, omnicomprehensive, and convincing explanation has not been yet found.

## 7. High-Mobility Group Box-1 and Microbiome

**Figure 1:**
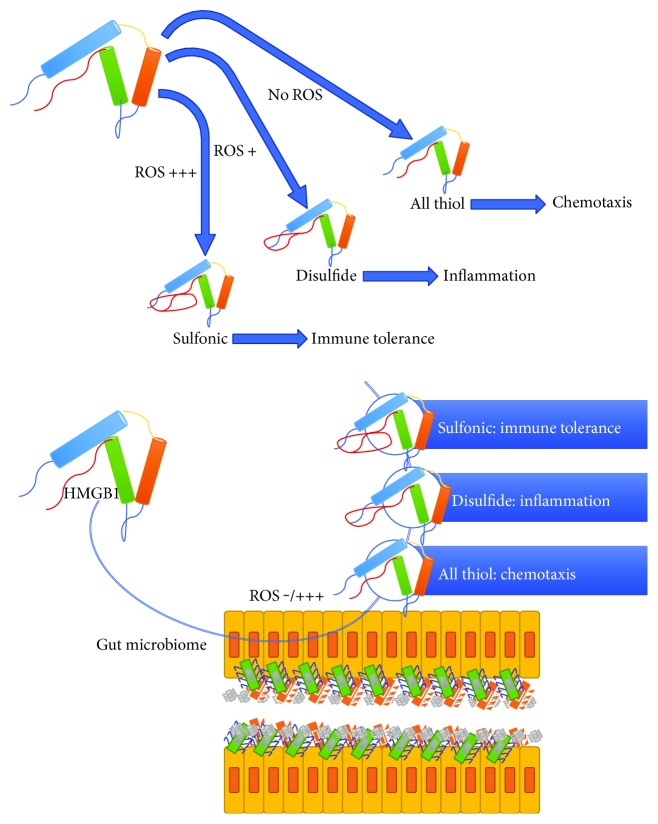
A schematic representation of the interaction and the crosstalk between HMGB1 and gut microbiome in RA pathogenesis. Depending on the oxidation/reduction status after the passage through the gut microbiome, HMGB1 can play several and different roles in RA initiation and maintenance.

Since LPS is one of the most important experimental activators of the HMGB1 pathway, it seems fair to assume that intestinal bacterial *flora* is involved in HMGB1 modulation. However, there is a lack of evidence about the crosstalk between HMGB1 and microbiome due, at least in part, to the difficulty of measuring tissue and fluid protein concentrations in its extracellular form. In fact, once released after cellular injury or activation, HMGB1 can be found in at least three conformations, depending on the oxidation/reduction status, and the commonly used experimental kits are not capable to detect all the conformations [58, 136, 137]. Furthermore, the complexity of the gut and the difficulty of obtaining reproducible data about the redox state of microbiota make it even more difficult task. However, HMGB1 surely plays a role in oral and intestinal homeostasis [155], and recent data demonstrated that this nuclear protein is involved in the inflammatory response of the gut and that the HMGB1 blockade is able to inhibit the LPS-induced injury by a TLR4-dependent mechanism [69]. In this model, TLR4 is considered a pivotal receptor for inflammation and the interaction between HMGB1 and TLR4 of mucosal tissue is important in inducing the intestinal inflammation. However, the inflammatory milieu is rich in oxidizing agents, and the HMGB1 translocation in this scenario could promote the structural modification of the protein. Furthermore, microbiome represents an important source of redox-based signals that modulate critical microbial and host cell functions [156–158]. Moreover, the microbiome modulates the redox status of the host by modifying the glutathione metabolism [159]. In addition, recent data obtained in both *in vivo* and *in vitro* models demonstrated a novel HMGB1-RAGE-mediated redox signaling pathway involved in intestinal inflammation induced by a liver dysfunction model [160]. As shown in Figure 1, HMGB1 conformational modulation depending on microbiome homeostasis could lead to different redox states and consequent activities. In this respect, the maintenance of a proper homeostasis of the microbiome may be important to prevent damage caused by HMGB1 overexpression.

## 8. Therapeutic Implications

A definitive treatment for all RA patients has not been discovered [23, 161–164]. A multitarget approach is required to better control the disease, and several pathways must be considered to completely treat RA. However, immunosuppressive drugs are not always sufficient [165–167]. For this reason, new therapeutical strategies are desirable and a better knowledge of HMGB1 interaction with microbiome in RA could provide new elements to achieve it. In this regard, a possible attempt could be the HMGB1 pathway blockade. In fact, several data demonstrated that, together with the commonly used monoclonal antibody-based therapies, monoclonal antibodies directed versus HMGB1 can protect against arthritis in experimental models [168, 169]. In particular, in two notably different models of arthritis, collagen-induced arthritis (CIA) and a genetic model of arthritis, Schierbeck and colleagues demonstrated that anti-HMGB1 monoclonal antibody administration significantly ameliorated the clinical courses in these experimental conditions. However, there is no evidence about the redox status and the possible role of microbiome in these models, and further data are needed to better understand the possible implications of an altered homeostasis of microbiome in HMGB1-dependent arthritis and in anti-HMGB1 therapy efficacy. Moreover, in a model where germ-free piglets were orally colonized with enteric bacterial pathogens, HMGB1 result significantly increased, suggesting that the upset balance of the microbiome can affect the HMGB1 pathway *equilibrium* [170]. Since the protein redox state can significantly modify the HMGB1 activity, a therapy capable of controlling the microbiome-oxidizing capacity could represent a new interesting approach. In this respect, probiotics need to be cited. Probiotic administration restores homeostasis of the gut microbiome and can have several beneficial effects [52]. Among the autoimmune disorders, RA seems to benefit from the probiotic therapy [54, 171]. Results obtained from animal models demonstrated that oral therapy with *Lactobacillus casei* ameliorated CIA by downregulating T helper 1 effector functions [172] and by reducing proinflammatory cytokines [173]. Also, data from humans have been achieved. In particular, in 46 patients with RA, *Lactobacillus casei* was orally administrated for 8 weeks and the disease activity score and serum proinflammatory cytokines were significantly decreased by the intervention [174]. In this setting, it is possible to speculate that homeostasis of microbiome could regulate HMGB1 activities in these patients. However, additional data are required to confirm this hypothesis.

## 9. Conclusions

RA is a chronic, harmful, and potentially severe disease for which there is no yet a decisive treatment. HMGB1 and microbiome alterations are involved in pathogenesis of RA, and the crosstalk between the protein and the microbiome deserves to be studied more carefully in order to offer a new therapeutic tool for patients with this serious disease.
